# New cut-off values for screening of trisomy 21, 18 and open neural tube defects (ONTD) during the second trimester in pregnant women with advanced maternal age

**DOI:** 10.1186/s12884-020-03464-z

**Published:** 2020-12-14

**Authors:** Yiming Chen, Xue Wang, Liyao Li, Sha Lu, Zhifen Zhang

**Affiliations:** 1 Department of Prenatal Diagnosis and Screening Center, Hangzhou Women’s Hospital (Hangzhou Maternity and Child Health Care Hospital), No. 369, kunpeng Road, Shangcheng District, Hangzhou Zhejiang, 310008 China; 2 Nanjing Medical University, Nanjing Jiangsu, 210029 China

**Keywords:** Advanced maternal age;alpha-fetoprotein, Down syndrome, Free beta human chorionic gonadotropin, Positive rate, Positive predictive value (PPV)

## Abstract

**Background:**

To determine whether advanced maternal age (AMA) causes changes in the maternal serum markers of Trisomy 21, 18 and open neural tube defects (ONTD) during the second trimester of pregnancy. Our research aims to develop new cut-off values for AMA in order to reduce the need for further invasive testing.

**Methods:**

This retrospective cohort study involved 12,739 pregnant women with AMA and 197,101 pregnant women with non-AMA. We then compared the two groups with respect to the positive rate and positive predictive value (PPV) of Trisomy 21, 18 and ONTD. Pregnant women with Trisomy 21, 18 and ONTD were diagnosed by karyotyping the amniotic fluid and by ultrasound diagnosis.

**Results:**

Compared to the non-AMA group, the multiple of the median (MOM) of free beta- human chorionic gonadotropin (free β-hCG), alpha-fetoprotein (AFP), and the risk value forTrisomy 21, were significantly higher in the AMA group (all *P* < 0.001). The positive rates of Trisomy 21, 18, and ONTD in the AMA group were significantly higher than those in the control group (all *P* < 0.001). In the AMA group, the PPVs for Trisomy 21 and other deformities were significantly higher (all *P* < 0.001), although the PPVs for Trisomy 18 and ONTD were similar to those of the non-AMA group. The area under the curve (AUC) values for the AMA group were higher than the non-AMA group, based on free β-hCG MoM, AFP MoM, and the risk value of Trisomy 21. The cut-off value for the risk value of Trisomy 21 was 1/172 for the AMA, group and 1/780 for the non-AMA group.

**Conclusions:**

The positive rates for Trisomy 21, 18 and ONTD, and the PPV for Trisomy 21 and other deformities were significantly higher in the AMA group. It is essential for pregnant women with AMA to be tested using appropriate cut-off values of serum markers screening for Trisomy 21 during the second trimester of pregnancy to improve the efficacy of prenatal screening and reduce the need for further invasive testing.

## Background

Trisomy 21, also referred to as Down’s Syndrome (DS) or the ‘congenital type’, and trisomy 18, which is also known as Edwards’ syndrome (ES), are the most common chromosomal abnormalities, with neonatal incidences of 1/800–1/600 [1] and 1/2600–1/2500 [2], respectively. Because these conditions both include additional chromosomal material, Trisomy 21 and 18 are characterized by irreversible mental retardation. The survivors of this condition do not generally have the ability to take care of themselves. Moreover, Trisomy 21 and 18 are the most common hereditary causes of low intelligence and account for 90% of all neonatal chromosomal diseases [1–3]. Open neural tube defects (ONTD) are considered to be serious congenital birth defects and generally occur before 4 weeks of pregnancy. Such defects arise because the neural tube fails to close. There are many forms of ONTD, including cranial dysraphism and spinal dysraphism; the former is fatal and results in abortion, infant death or still birth. In contrast, spinal dysraphism causes symptoms of paralysis and incontinence [4]. Previous research showed that ONTD affects 1.2 per 1000 pregnancies worldwide [5].

Previous studies have shown that women of advanced maternal age (AMA) have a higher incidence of Trisomy 21 [6, 7], although the precise mechanisms underlying these observations remain unclear. Although a range of biomarkers exist for the detection of ONTD and Trisomy 21, there is an increasing shift towards the use of cell-free DNA (cfDNA) for the detection of fetal aneuploidies. It is possible that the development of such tests may have significant impact upon the ways in which we screen for ONTD and Trisomy 21. Data also appear to suggest that the levels of serum AFP in the pregnant women may represent a potential standalone screen for ONTD only [8]. In a previous study, AMA led to an increase in sister kinetochore separation, rotated bivalents and merotelic attachments, and revealed multiple age-related changes in chromosome architecture, thus, providing an explanation for the increased levels of oocyte aneuploidy with AMA [9]. Many countries now recommend that pregnant women with a maternal age of 35 years or more should undergo interventional prenatal diagnosis during the second trimester in order to avoid fetuses being born with chromosomal abnormalities and ONTD [10, 11]. However, some studies have reported that the prenatal screening of serum Trisomy 21 markers in pregnant women of AMA could significantly reduce the probability of interventional prenatal diagnosis [12, 13]. Indeed, Been et al. suggested that maternal serum screening and ultrasonography, when carried out in the second trimester, resulted in more judicious use of amniocentesis and chorionic villus sampling [14]. However, very few studies have attempted to investigate the precise association between AMA and ONTD.

We conducted a retrospective cohort study and carried out screening for key maternal serum biomarkers in 12,739 pregnant women with AMA and 197,101 pregnant women with non-AMA. We then compared the two groups with respect to the positive predictive value (PPV) of Trisomy 21, 18 and ONTD. Our aim was to investigate the reliability of maternal serum screening for pregnant women with AMA during the second trimester of pregnancy, and to identify new cut-off values for the Trisomy 21, Trisomy 18 and ONTD to reduce the need of amniocentesis.

## Methods

### Study population

This was a cohort study that was conducted in Hangzhou Women’s Hospital, China. The Hangzhou government implements free prenatal screening involving AFP and free β-hCG tests for patients registered locally and the floating who have lived in the area for more than 6 months. We recruited 209,840 pregnant women from Hangzhou between January 2015 and October 2018. If the expected maternal age at birth was 35 years or older, then the gravidas were allocated to an AMA group (12, 739 cases; 6.07%) or a non-AMA group (197,101 cases; 93.97%).

Pregnant women were included in the study if they were between15 weeks and 20 weeks 6 days in gestational age, had a singleton pregnancy, and agreed to be screened for Trisomy 21, 18 and ONTD during the second trimester. Pregnant women were excluded for the following reasons: multiple pregnancy, smoking, diabetes, a history of chromosomal abnormalities and congenital abnormalities, infants conceived in vitro, and a range of pregnancy-related diseases, including hypertensive disorders of pregnancy, gestational diabetes mellitus, and intrahepatic cholestasis of pregnancy.

Trisomy 21 and 18were diagnosed by the chromosomal karyotyping of amniotic fluid cells while ONTD was diagnosed by ultrasound. Community nurses also performed a follow-up for each pregnant woman just 1 year after birth to check for aneuploidy and defects. Chromosomal examinations were also carried out for all stillbirths.

### Measurements

Fasting venous blood samples were drawn from each pregnant woman and the samples were separated for 30 min and centrifuged at 2000 g for 10 min. Separated sera were then placed in a refrigerator at 2–8 °C and sent for laboratory testing. A 1235 Automatic Immunoassay System (PerkinElmer, Shelton, USA) was used to measure free beta-human chorionic gonadotropin (free β-hCG) and alpha-fetoprotein (AFP) and the assays were carried out in accordance with standardized protocols. An ultrasound system (VolusonE8, GE) was used for prenatal diagnosis.

The risk values of Trisomy 21, 18 and ONTD were calculated by Life Cycle 4.0 software (Perkin Elmer, Wallac, US), taking into account maternal age, gestational age and maternal weight [15]. The cut-off values were as follows: Trisomy 21 ≥ 1:270; Trisomy 18 ≥ 1: 350, AFP MoM ≥2.50, high risk of ONTD [16]. Pregnant women with a high risk of Trisomy 21 and Trisomy 18 were advised to undergo karyotype analysis using the amniotic fluid cells in order to confirm the diagnosis. Women associated with a high risk of ONTD were advised to undergo ultrasound diagnosis. The measured AFP and free β-hCG levels were expressed as MOM values. These were adjusted by gestational age and maternal weight. If the menstrual period was regular, then gestational age was determined by the last menstrual period. Otherwise, the double-top diameter was used to confirm the gestational age. If pregnant woman had both a top arm diameter and a double top diameter, then we mainly used the top arm diameter to determine gestational age.

### Statistical analysis

All statistical analyses were performed using IBM SPSS Statistical software (version 21.0; Armonk, N.Y.; USA). The one-sample Kolmogorov-Smirnov test was used to test raw data for normality. Tests showed that maternal age, maternal weight, and gestational age, all exhibited a skewed distribution. These data were then expressed as medians and percentiles [M(P_2.5_-P_97.5_)]. The Mann-Whitney U test was used to make comparisons between two groups. Comparison of PPV involved the *χ*^*2*^ test or a continuous correction *χ*^*2*^ test. A *P* value < 0.05 was considered to be statistically significant.

## Results

Maternal age, maternal weight and gestational age in the AMA group were all significantly higher than in the non-AMA group (Z = 189.464, *P* < 0.001; Z = 4.883, *P* < 0.001; Z = 2.261, *P* < 0.001, respectively; Table 1). The multiple of the median (MoM) value for free β-hCG, AFP, along with the risk value of Trisomy 21, were all significantly higher in the AMA group than in the non-AMA group (Z = 6.076, *P* < 0.001; Z = 21.964, *P* < 0.001; Z = 98.884, *P* < 0.001, respectively; Table 2). The positive rate for cases with a high risk of Trisomy 21, 18 and ONTD in the AMA group were 19.55% (2490/12739), 1.30% (165/12739), and 0.68% (87/12739), respectively. Those rates were all significantly higher than in the non-AMA group (5.09% (10,037/197101), 0.28% (559/197101), 0.43% (839/197101)(*χ*^*2*^ = 4453.316, *P* < 0.001; *χ*^*2*^ = 356.143, *P* < 0.001; *χ*^*2*^ = 18.027; *P* < 0.001, respectively; Table 3).

**Table 1:** Basic demographic data of each group

Group	N	maternal age (years)	gestational age (days)	maternal weight (kg)
Non-AMA	197,101	28.41(21.54 ~ 34.43)	118.00(108.00 ~ 134.00)	54.50(43.00 ~ 74.60)
AMA	12,739	36.87(35.05 ~ 42.39)	118.00(109.00 ~ 134.00)	57.50(45.00 ~ 76.50)
*z*		189.464	4.883	2.261
*P*		< 0.001	< 0.001	< 0.001

*AMA* advanced maternal age

**Table 2:** Comparation of maternal serum markers of Trisomy 21 and 18 risk value

Group	N	AFP (KU/L)	AFP (MoM)	Free β- hCG (μg/L)
Non-AMA	197,101	37.2(19.20 ~ 75.50)	0.98(0.54 ~ 1.87)	14.30(4.45 ~ 57.20)
AMA	12,739	37.9(19.30 ~ 77.50)	1.05(0.56 ~ 1.99)	14.30(4.27 ~ 59.75)
*z*		6.058	21.964	1.111
*P*		< 0.001	< 0.001	0.911
Group		Free β- hCG (MoM)	Risk value of Trisomy 21	Risk value of Trisomy 18
Non-AMA		0.99(0.33 ~ 3.60)	1/3934(1/133 ~ 1/33336)	1/46055(1/2477 ~ 1/100000)
AMA		1.02(0.33 ~ 3.97)	1/1003(1/25 ~ 1/9648)	1/11686(1/624 ~ 1/44622)
*z*		6.076	98.885	124.780
*P*		< 0.001	< 0.001	< 0.001

*AMA* advanced maternal age, *AFP* alpha-fetoprotein, *free β-hCG* free beta human chorionic gonadotropin, *MoM* multiple of the median

**Table 3:** Comparation of results of prenatal screening

Group	High risk			Low risk			Sum
	Trisomy 21	Trisomy 18	ONTD	Trisomy 21	Trisomy 18	ONTD	
Non-AMA	10,037 (5.09)	559 (0.28)	839 (0.43)	187,064 (94.91)	196,542 (99.72)	196,262 (99.57)	197,101
AMA	2490 (19.55)	165 (1.30)	87 (0.68)	10,249 (80.45)	12,574 (98.70)	12,652 (99.32)	12,739
Sum	12,527 (5.97)	724 (0.35)	926 (0.44)	197,313 (94.03)	209,116 (99.65)	208,914 (99.56)	209,840

*AMA* advanced maternal age, *ONTD* Open neural tube defects

Table 4 shows that the PPVs for Trisomy 21, 18, ONTD, and other deformities in the AMA group were 2.20‰ (28/12739), 0.08‰ (1/12739), 0.08‰ (1/12739), and 15.23‰ (194/12739), respectively. In the non-AMA group, the PPVs for Trisomy 21, 18, ONTD, and other deformity were 0.24‰ (48/197101), 0.08‰ (15/197101), 0.07‰ (14/197101), and 8.36‰ (1647/197101), respectively. The PPV for Trisomy 21 and other deformities were significantly different when compared between the AMA and non-AMA groups (*χ*^*2*^ = 126.245, *P* < 0.001; *χ*^*2*^ = 50.329, *P* < 0.001); however, the PPVs for Trisomy 18 and ONTD were not significantly different.

**Table 4:** Comparation of PPV of Trisomy 21, 18, ONTD and other deformity (n ‰)

Group	n	High risk				Low risk				Sum			
		Trisomy 21	Trisomy 18	ONTD	Other deformity	Trisomy 21	Trisomy 18	ONTD	Other deformity	Trisomy 21	Trisomy 18	ONTD	Other deformity
Non-AMA	197,101	24(0.12‰)	10(0.05‰)	4(0.02‰)	202(1.02‰)	24(0.12‰)	5(0.03‰)	10(0.05‰)	1445(7.33‰)	48(0.24‰)	15(0.08‰)	14(0.07‰)	1647(8.36‰)
AMA	12,739	20(1.57‰)	1(0.08‰)	0	54(4.24‰)	8(0.63‰)	0	1(0.08‰)	140(10.99‰)	28(2.20‰)	1(0.08‰)	1(0.08‰)	194(15.23‰)
Sum		44	11	4	256	32	5	11	1585	76	16	15	1841

*AMA* advanced maternal age, *ONTD* Open neural tube defects; Other deformity, chromosomal or structural abnormalities other than Trisomy 21, 18, and ONTD; PPV, positive predictive value

**Fig. 1:**
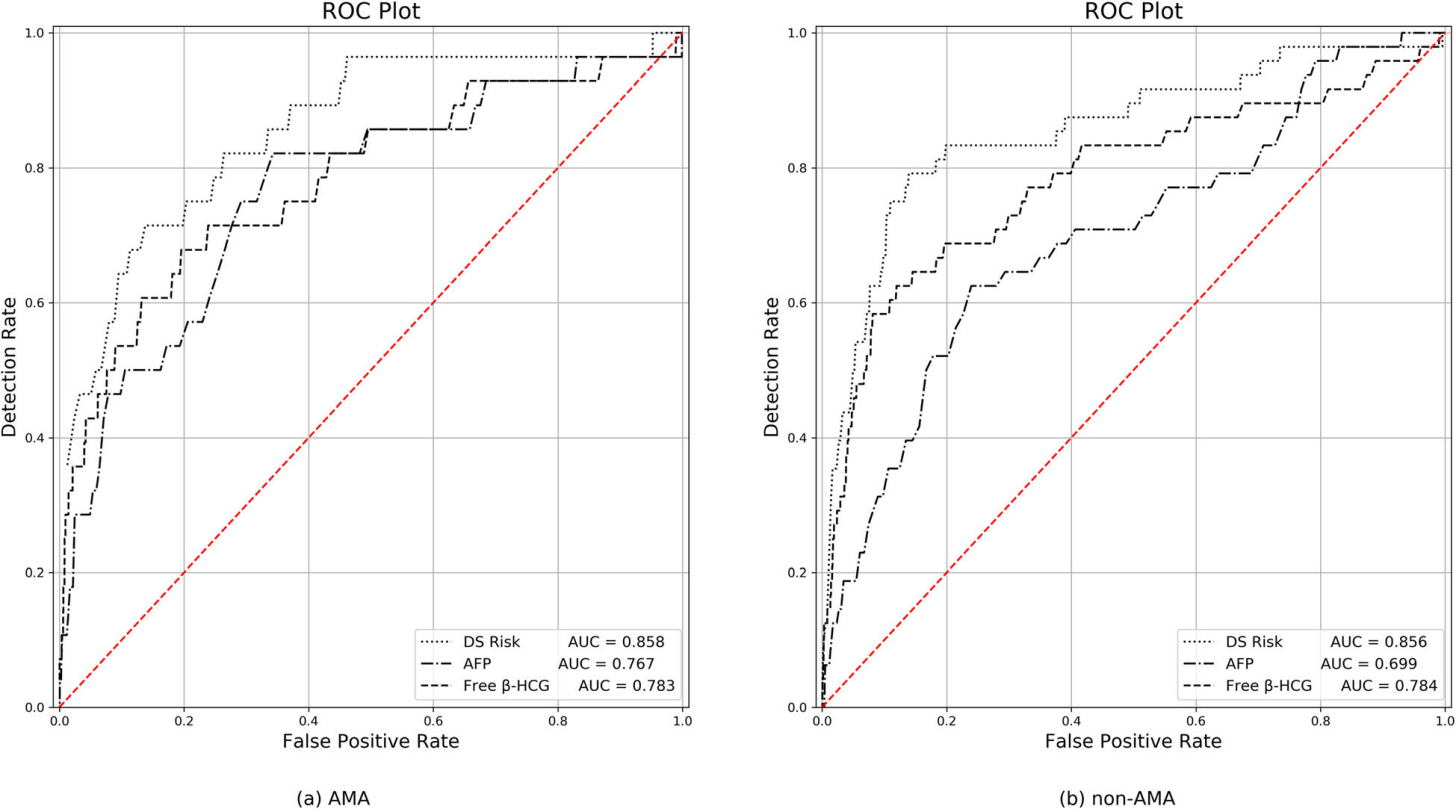
AUC of maternal free β-hCG MoM, AFP MoM and risk value of Trisomy 21 for Trisomy 21 in ROC curves. **a** AUC of AMA; **b** AUC of non-AMA

In the AMA group, the area under curve (AUC) was 0.783, 0.767, and 0.858, for the PPVs for maternal free β-hCG MoM, AFP MoM, and the risk value of Trisomy 21, respectively. When the cut-off values were 1.725 MoM, 0.925 MoM, and 1/172, respectively, the corresponding sensitivities were 0.679, 0.821, and 0.714, respectively (Table 5; Fig. 1a). In the non-AMA group, the AUC was 0.784, 0.699 and 0.856 for maternal free β-hCG MoM, AFP MoM, and the risk value of Trisomy 21, respectively. When the cut-off values were 2.055 MoM, 0.795 MoM, and 1/780, respectively, the corresponding sensitivities were 0.675, 0.675, and 0.792, respectively (Table 5; Fig. 1b).

**Table 5:** Predictive values of maternal free β- hCG, AFP MoM, and risk value of Trisomy 21 for Trisomy 21

Group or item	N	AUC	95%CI	*P*	cut-off	Sensitivity	Specificity	Youden index
Non-AMA	197,101							
free β- hCG (MoM)		0.784	0.703–0.865	< 0.001	2.055	0.675	0.881	0.506
AFP (MoM)		0.699	0.618–0.779	< 0.001	0.795	0.675	0.760	0.385
Risk value of Trisomy 21		0.856	0.792–0.920	< 0.001	1/780	0.792	0.862	0.654
AMA	12,739							
free β- hCG (MoM)		0.783	0.680–0.885	< 0.001	1.725	0.679	0.805	0.483
AFP (MoM)		0.767	0.668–0.865	< 0.001	0.925	0.821	0.656	0.477
Risk value of Trisomy 21		0.858	0.780–0.936	< 0.001	1/172	0.714	0.866	0.580

*AMA* advanced maternal age, *AFP* alpha-fetoprotein, *free β-hCG* free beta human chorionic gonadotropin, *MoM* multiple of the median

In Table 6, the sensitivity, PPV and false positive rate of Trisomy 21 screening for pregnant women in the AMA group were all higher than those in the non-AMA group.

**Table 6:** Predicted value and diagnostic value of prenatal screening for Trisomy 21 (%)

Group	Screening for disease	Sensitivity	Specificity	Positive predictive value	Negative predictive value	False positive rate	False negative rate
AMA	Trisomy 21	71.43	80.56	0.80	99.92	19.43	28.57
Non-AMA	Trisomy 21	50.00	94.92	0.24	99.99	5.08	50.00

## Discussion

Collectively, the liberalization of the second child policy, and the increasing pressure to work, is leading towards an era of pregnancies in women of AMA. In the USA, the proportion of pregnancies in women of AMA was approximately 5% in the 1970s, but rose to 14%by 2002. Furthermore, AMA pregnancies accounted for more than 50% of cases involving Trisomy 21 in 2002 [17]. In our study, we identified 12,739 pregnant women with AMA, accounting for 6.07% of our study population. The PPV for Trisomy 21 was 2.20‰, which was higher than that described in other recent reports (5.46% AMA and a PPV of 129 per million for Trisomy 21) [18, 19]. Because there are only a small number of medical institutions that are able to carry out prenatal diagnosis, not all pregnant women are able to undergo such testing, or miss the time window to undergo these important tests. Furthermore, some pregnant women are afraid and fail to undergo prenatal diagnosis because they are concerned about the 1.21% abortion rate that is associated with amniocentesis [20]. The American College of Obstetricians and Gynecologists recommend that regardless of the age of pregnant women, the serum markers for selected aneuploidy conditions should be screened in pregnancy before further interventional prenatal diagnosis [21]. In Tunisia, the guidelines are that serum marker testing should be offered to all patients, including women of AMA, and that routine amniocentesis for women of AMA should be avoided [22]. Collectively, these studies demonstrate the necessity for serum prenatal screening for Trisomy 21 in women of AMA.

Because factors related to maternal age are included in the risk calculation for Trisomy 21 and 18 [23], the MoM value for free β-hCG and AFP, and the risk values for Trisomy 21, in women of AMA were significantly higher than in the non-AMA group (all *P* < 0.001). Furthermore, the positive rates for a high risk of Trisomy 21, 18and ONTD in the AMA group were significantly higher than in the non-AMA group (all *P* < 0.001). The positive rates for a high risk of Trisomy 21 in the AMA and non-AMA groups were 19.55 and 5.09% respectively. These data were similar to the data reported previously by Gyselaers et al. [24] study. We found that the PPVs for Trisomy 21, 18, ONTD, and other deformities in women of AMA were higher than in the non-AMA group. The PPVs for Trisomy 21 and other deformities were both significantly different when compared between the two groups (all *P* < 0.001).

The PPV for other deformities in women of AMA were 15.23‰ (194/12739) in our study, including 26 chromosomal abnormalities with a PPV of 2.04‰ (26/12739). These results suggested that maternal serum markers are not only useful for screening Trisomy 21, 18, and ONTD, but are also useful for screening other diseases, including chromosomal abnormalities. The PPVs for Trisomy 18 and ONTD was not significantly different when compared between the two groups, which may be related to the low number of ES and ONTD in the AMA group, and therefore, needs further verification. Resta et al. previously found that offering amniocentesis to women who were 35-years-of-age and above would result in one in seven of the pregnant women subsequently undergoing amniocentesis [17]. Based on likelihood ratios, using AMA as a screening strategy for Trisomy 21 is significantly inferior to a combination of serum and sonographic screening. Therefore, it is necessary to strengthen the management of high-risk and low-risk pregnant women during the late stages of prenatal screening, particularly in terms of the sonographic screening strategies.

We found that the combination of serum screening with maternal age in the second trimester was more effective than using maternal age alone to screen for Trisomy 21. Prenatal ultrasonography for soft markers of chromosomal aneuploidy, accompanied by maternal serum biochemical screening tests, should be evaluated during the decision-making process when considering genetic amniocentesis in women of AMA. Patients should be educated by recommending women of AMA to be informed of both screening and amniocentesis options [25, 26]. We also found that the AUCs were 0.783, 0.767 and 0.858, respectively, for the PPVs of free β-hCG MoM, AFP MoM, and the risk value for Trisomy 21 in women of AMA. These PPVs were significantly higher than the corresponding AUCs for the non-AMA group (0.784, 0.699, and 0.856). With a similar sensitivity, the cut-off values for the risk value of Trisomy 21 was 1/172, much higher than the risk value of the non-AMA group (1/780). This indicated that if the cut-off value for the non-AMA group was applied to judge all pregnant women, then between 1/172 and 1/780 pregnancies would undergo amniocentesis, thus, causing interventional prenatal diagnosis that was unnecessary.

## Conclusions

We found that with regards to maternal serum screening, the positive rate for a high risk in women of AMA was significantly higher than that of younger pregnant women. The PPVs for Trisomy 21 and other malformations were both higher in women of AMA than the non-AMA group. It is essential that we significantly reduce the number of unnecessary interventional prenatal diagnoses and improve the efficacy of prenatal screening.

## Data Availability

All data generated or analyzed during this study are included in the supplementary file and this published article.

## Abbreviations

AMA: Advanced maternal age
NT: Nuchal translucency
AFP: Alpha-fetoprotein
free β-hCG: Free beta human chorionic gonadotropin
MoM: Multiple of the median
DS: Down’s Syndrome
ES: Edwards’ syndrome
ONTD: Open neural tube defects
AUC: Area under curve
PPV: Positive predictive value
